# Development of a Temperature Distribution Measurement System for Transmission Oil for Transportation Equipment

**DOI:** 10.3390/s23125499

**Published:** 2023-06-11

**Authors:** Shumpei Funatani, Ryoga Takei, Yusaku Tsukamoto

**Affiliations:** Department of Mechanical Engineering, University of Yamanashi, Yamanashi 400-8510, Japan

**Keywords:** LIF (laser-induced fluorescence), two-wavelength LIF, temperature measurement, oil flow, optical sensor, phosphor

## Abstract

In this study, an optical sensor using thermo-sensitive phosphor and its measurement system for visualizing and measuring the temperature distribution in an arbitrary cross-section of transmission oil using one type of phosphor, whose peak wavelength changes with temperature, is proposed. Because the intensity of the excitation light is gradually attenuated by the scattering of the laser light owing to microscopic impurities in the oil, we attempted to reduce the scattering effect by increasing the excitation light wavelength. Therefore, Pyrromethene 597 was selected as the optical sensor using thermo-sensitive phosphor, and a DPSS (Diode Pumped Solid State) laser with a wavelength of 532 nm was used as the excitation light. Using this measurement system, we measured the temperature distribution of a vertical buoyant jet of transmission oil and validated the measurement method. In addition, it was shown that this measurement system could be applied to the measurement of the temperature distribution in transmission oil with cavitation foaming.

## 1. Introduction

A transmission system used to transmit the power of an automobile engine can efficiently transmit the output from the engine to the wheels by meshing several gears. Owing to the nature of transmission, the lubrication of the gear teeth during meshing is important. Therefore, a lubricant, transmission oil, is required to perform functions such as improving power transmission efficiency and durability. Transmission oil has a lifespan in which it can perform its normal functions, and various factors contribute to the aging of the oil.

It has been reported that cavitation occurs in the oil during transmission, causing a popping sound owing to the bursting of bubbles. The cavitation in transmission oil is of particular concern because it degrades the oil, erodes gears, damages transmission, and degrades the transmission efficiency owing to the reduced lubrication characteristics. If the bubbles are not eliminated, it is possible that the transmission oil will oxidize and the bubbles will travel along the lubrication path, making it difficult to maintain a normal oil film thickness [1]. When high pressure is applied to a bubble, the temperature of the gas in the bubble increases because of adiabatic compression, and heat is transferred to the transmission, increasing the temperature of the transmission oil. Similarly, adiabatic compression occurs when bubbles burst instantaneously owing to cavitation, resulting in a temperature increase in the transmission oil. As mentioned above, the temperature increase in the transmission oil causes deterioration. In particular, the effect of a local temperature rise, such as cavitation, on the transmission oil requires a detailed investigation.

As optical sensors for flow diagnostics with temperature distribution, there are precedents for two-dimensional and three-dimensional temperature distribution measurements using the temperature-sensitive liquid crystal method [2] and the two-color LIF (laser-induced fluorescence) method [3,4,5,6,7]. The two-color LIF method was applied to clarify the mechanism of the Rayleigh–Bénard convection phenomenon [8,9,10,11,12]. These measurement methods utilize the temperature dependence of the emission wavelength and the intensity of phosphors. The two-color LIF method has also been used to measure the temperature distribution of airflow [13] and oil droplets [14,15]. However, since the particle diameter of the thermosensitive liquid crystal is approximately 10 μm, fluid temperature measurements of micrometer-order using this thermosensitive liquid crystal are difficult. Therefore, the temperature-sensitive liquid crystal method is unsuitable for evaluating the cavitation phenomena, whereas the LIF method is suitable for measuring the oil temperature around cavitation bubbles.

The LIF method has other advantages. Although the fluid to be measured is colored, no issues are observed with temperature measurement if the color of the phosphor can be captured. In addition, the temperature measurement can still be measured when phosphor paint is added to the transmission oil, provided the phosphor coloration is not disturbed.

Based on the above, it can be concluded that the two-color LIF method can be used to visualize the temperature distribution for temperature measurements around bubbles, such as cavitation, on a fine scale. Moreover, the oil used in this study is an organic solvent; hence, water-soluble phosphors cannot be used. However, concerns such as the change in oil characteristics owing to a mixture of two types of phosphors and the transmission oil being composed of additives instead of single substances were raised. Oil components are generally colored fluids with several additives, which cause the attenuation of laser light used for visualization and measurement.

The purpose of this study is to propose a method of verifying the temperature changes in transmission oil caused by cavitation. This is achieved by constructing a system that can visualize the temperature distribution of an arbitrary section of the transmission oil using a single thermo-sensitive phosphor whose peak wavelength changes with temperature.

## 2. Measurement Methods

### 2.1. Characteristics of Phosphor Material as Sensors for Temperature Measurement

LIF (laser-induced fluorescence) [3,4,5,6,7] is a measurement method that uses laser light to generate the fluorescence of specific molecules and measure scalar quantities such as the concentration, temperature distribution, and physical quantity of specific substances from the intensity of emission at a specific wavelength. The PIV (particle image velocimetry) method is frequently used in conjunction with the LIF method. In PIV, minute tracer particles are mixed with fluid and momentarily irradiated with a sheet of light to obtain two consecutive images of the particles at two different intervals [16]. Furthermore, this method obtains the flow from the displacement vectors of the particles moving between images. The PIV and LIF methods are closely related, and share several points of reference in the image processing process and the construction of experimental systems.

In contrast to the abovementioned LIF method, the two-color LIF method uses two types of thermo-sensitive phosphors to achieve a higher temporal resolution and accuracy. The basic principle of this method is to simultaneously excite two phosphors with different fluorescence characteristics and wavelengths to obtain a scalar quantity that can be measured from the ratio of the fluorescence intensity of each thermo-sensitive phosphor.

Factors that affect fluorescence and quenching when using the LIF method include the following:

(1) pH

When the absorbance or absorption spectrum of a fluorophore molecule changes with the solvent pH, the fluorescence intensity also changes.

(2) Solution polarity

The absorption spectra change depending on the polarity and state of the liquid solvent.

(3) Concentration quenching

The energy exchanged between molecules is not emitted as fluorescence because the distance between the phosphor molecules in the solution decreases as the concentration of the phosphor molecules increases.

(4) Temperature quenching

The fluorescence intensity decreases owing to the loss of energy by molecular collision, because molecular motion becomes more active as the temperature rises.

(5) Photo-quenching

Quenching is a phenomenon in which fluorescent molecules are excessively activated when the intensity of the excitation light is excessively high, causing irreversible changes [3,4,5,6,7].

**Figure 1:**
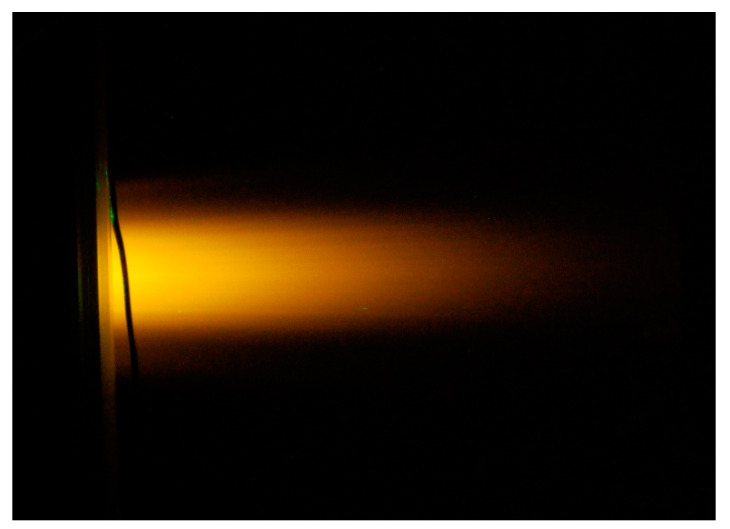
Attenuation of laser intensity in transmission oil flow.

When using the colored-fluid LIF method, it is necessary to first estimate the laser light intensity, because it may be attenuated in the optical path from the laser irradiation surface to the area to be analyzed. When measuring a surface, a large change in the intensity of the laser beam within the measurement area can reduce measurement accuracy (Figure 1).

When a phosphor material is irradiated with excitation light, the electron distribution in the molecule changes, and its energy level transitions from the ground to excited level. This is called an electronic transition. Part of the excited energy is transferred to the kinetic energy of the surrounding molecules via vibration and rotation (nonradiative decay), whereas the remaining energy is emitted as photons (radiative decay). This radiative decay is either fluorescent or phosphorescent. The optical properties of phosphors include fluorescence and phosphorescence, and this study deals only with fluorescence.

In phosphor, the light energy I (W⁄m^3^) emitted per unit time by a unit microvolume of phosphor is proportional to the number of molecules absorbing photons of excitation light in a unit volume per unit time, and is expressed by Equation (1):

(1) $$I=I_{0}C\phi \varepsilon$$

where *I_0_* (W/m^2^) is the excitation light flux incident on the microvolume, *C* (g/m^3^) is the concentration of the fluorophore, *ϕ* is the quantum yield, which indicates the fraction of absorbed excitation light that contributes to fluorescence emission, and *ε* (m^2^/g) is the absorption coefficient, which indicates the ratio of light intensity absorbed when excitation light passes a unit length of solution with unit concentration to the incident excitation light intensity. The light flux I’_0_ (m^2^/g) is the absorption coefficient of the light flux.

(2) $$I_{0}=I_{0}^{\prime }\exp\left(-\varepsilon xC\right)$$

Equation (2) is known as the Beer–Lambert law, which states that incident light is absorbed and attenuated during its passage through a solution. Therefore, the emission energy of fluorescence owing to the excitation light passing through a finite volume can be expressed using Equations (1) and (2) as follows:

(3) $$I=I_{0}^{\prime }C\phi \varepsilon \exp\left(-\varepsilon xC\right)$$

where the quantum yield *ϕ* is temperature-dependent and generally decreases with increasing temperature. Moreover, the fluorescence intensity is a function of temperature when *I_0_* and *C* are constants because the temperature dependence of the absorption coefficient *ε* is minimal. This decrease in the fluorescence intensity with increasing temperature is referred to as temperature quenching. By applying a function showing the dependence of the fluorescence intensity on the temperature of the measured image, the temperature distribution can be visualized [5,6].

In addition to temperature quenching, the following factors can cause phosphor quenching:

(1) High phosphor concentrations (concentration quenching).
(2) The presence of oxygen and ions (chlorine and various metal ions) in the solution (chemical quenching).
(3) Excessive excitation of light intensity (quenching caused by excessive excitation light intensity).

Therefore, the effects of these quenching factors must be considered. As a countermeasure against concentration quenching, the excitation light intensity, distance from the irradiated surface to the measurement area, and uniformity of fluorescence in the measurement area were investigated first, and an appropriate phosphor concentration was set. As for quenching owing to oxygen and ions, no data exist for transmission oil. Therefore, experimental tests were conducted to confirm that there was no chemical quenching.

The conventional two-color LIF method [3,4,5,6,7] focused on the temperature characteristics of the abovementioned phosphors for two types of phosphors. When phosphors A and B are used, the light energy emitted per unit time per unit microvolume of phosphor is expressed by the following equation:

(4) $$I_{A}=I_{0}^{\prime }C_{A}\phi_{A}\varepsilon_{A}\exp\left(-\varepsilon_{0}xC_{0}\right)$$

(5) $$I_{B}=I_{0}^{\prime }C_{B}\phi_{B}\varepsilon_{B}\exp\left(-\varepsilon_{0}xC_{0}\right)$$

Because the two-color LIF method uses the ratio of the two types of fluorescence, the ratio *R_I_* of the fluorescence is given as follows:

(6) $$R_{I}=\frac{I_{A}}{I_{B}}=\frac{I_{0}^{\prime }C_{A}\phi_{A}\varepsilon_{A}\exp\left(-\varepsilon_{0}xC_{0}\right)}{I_{0}^{\prime }C_{B}\phi_{B}\varepsilon_{B}\exp\left(-\varepsilon_{0}xC_{0}\right)}=\frac{C_{A}\phi_{A}\varepsilon_{A}}{C_{B}\phi_{B}\varepsilon_{B}}$$

**Figure 2:**
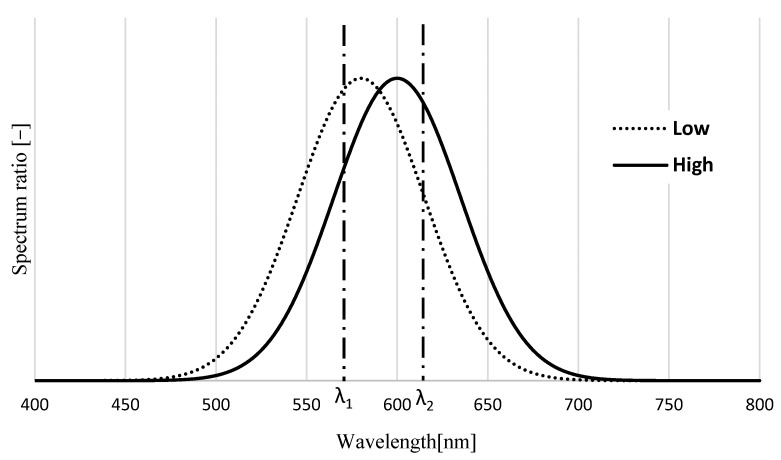
Temperature dependence of the fluorescence spectrum.

Equation (6) shows that the excitation light intensity does not affect the luminance ratio. Therefore, if *C_A_*_,*B*_ and *ε_A_*_,*B*_ are maintained at a constant, the luminance ratio will have an accurate dependence on temperature by considering two wavelengths of phosphor with temperature dependence. However, the effect of quenching is different for each phosphor, and this is one of the reasons for temperature measurement errors. Recently, it was found that the peak wavelength of the fluorescence spectrum of several kinds of phosphors has a temperature dependence. As shown in Figure 2, when the peak wavelength of the fluorescence spectrum has a temperature dependence, the fluorescence intensity ratio of the two wavelengths has a temperature dependence as in the two-color LIF method.

In this study, we named it the two-wavelength LIF method. The fluorescence intensity ratio is expressed in Equation (7).

(7) $$R_{\lambda }=\frac{I_{\lambda 1}}{I_{\lambda 2}}$$

where *I_λ1_* and *I_λ2_* are the luminance values at *λ_1_* and *λ_2_*, respectively. The luminance at *λ_1_* at low temperature is *I_L1_* and *I_L2_* at *λ_2_*. Similarly, *I_H1_* and *I_H2_* represent the luminance at *λ_1_* and *λ_2_*, respectively, at high temperature. Therefore, Equation (7) can be expressed as *I_L1_/I_L2_* and *I_H1_/I_H2_* (Figure 2).

The temperature dependence of the phosphor substance is not considered when using the ratio of luminance between two wavelengths; however, it is calculated as the ratio between specific wavelengths. Therefore, only one type of phosphor can be used, and the temperature measurement accuracy can be expected to improve because it is less affected by the excitation light intensity.

### 2.2. Measurement of Temperature Using the LIF Method

**Figure 3:**
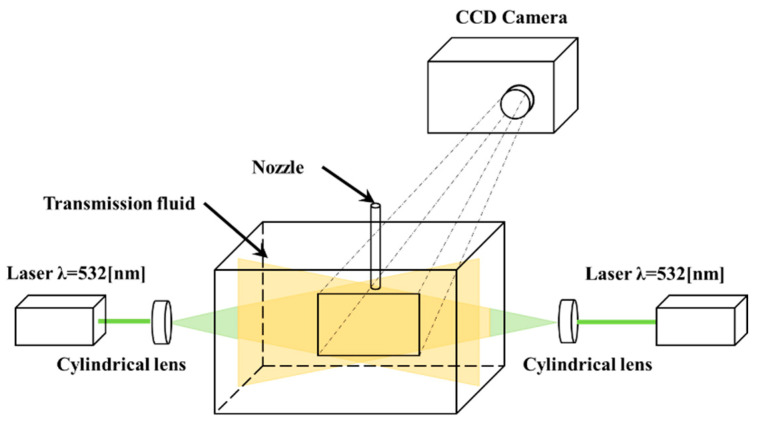
Experimental setup.

Figure 3 shows the experimental apparatus used to evaluate the temperature characteristics of the phosphor. The experimental apparatus comprised a water tank, temperature maintenance device, jet generator, laser, digital camera, agitator, and jigs. A plano-convex cylindrical lens was installed between the laser and water tank to facilitate the irradiation of the laser beam in the form of a sheet. In addition, a dark room was reproduced to improve the imaging accuracy during the experiments. The temperature of the transmission oil was regulated using a coil heater controlled by a PID controller.

The laser light was scattered by microscopic impurities in the oil, which caused gradual attenuation of the excitation light intensity. This attenuation is greater when the wavelength of the laser beam is shorter. Therefore, it is desirable to increase the laser beam wavelength. However, because the absorption wavelength of phosphors is shorter than their fluorescence wavelength, there are few phosphor selections [17,18]. Therefore, in this study, two DPSS lasers (Viasho Technology VA-532, wavelength: 532 nm, laser power: 100 mW) were used as the light source. The laser was a continuous light and no timing adjustment was performed. For the phosphor, Pyrromethene 597 was selected, which exhibits excitation characteristics as a light source with a wavelength of 532 nm [19]. There are no verified examples of the dispersibility or temperature dependence of Pyrromethene 597 in transmission oil. Pyrromethene 597 was mixed in transmission oil at a concentration of 10.77 mg/L and heated from 25 °C to 80 °C while being stirred in a container. Three images of the measurement area were obtained every 5 K increase in the transmission oil temperature, the luminance of each image was quantified, and the relationship between the average luminance of the measurement area and the temperature was investigated. The spectral distribution of fluorescence wavelengths was measured using a spectrometer (Hopoocolor HPCS-300, wavelength: 350–780 nm). In this study, we focused on the fact that the fluorescence wavelength of some phosphors changes in a temperature-dependent manner, and measured the temperature by acquiring the change in the fluorescence wavelength as the luminance ratio between two specific wavelengths. If Pyrromethene 597 exhibits temperature dependence, the temperature can be determined by measuring the luminance ratio of the Green image (wavelength: 550–575 nm) and Red image (wavelength: 580–635 nm) images in the obtained color image using a digital SLR camera (Nikon D40X, sensitivity: ISO1600, resolution: 3872 × 2592 pixels). Digital SLR cameras are equipped with a high-pass filter (λ > 550 nm) to block the wavelength of the DPSS laser (532 nm). The advantage of using a single-color camera is that the phase difference of a few pixels at the gas–liquid interface is reported to affect the accuracy of the measurement when measuring the temperature around cavitation bubbles, which is the objective. This will improve the measurement accuracy and simplify the measurement process. Assuming that the phosphors in the oil are sufficiently excited, the temperature can be calculated without being affected by the fluctuations in the fluorescence intensity of the phosphors. This can be achieved by calculating the fluorescence intensity ratio, including when the bubbles are mixed in the oil or when the laser beam output fluctuates. Hence, the visualization of the temperature distribution is possible through image analysis and processing in an arbitrary range of fluids with different temperature distributions, such as transmission oils.

## 3. Results

### 3.1. Temperature Dependence of Pyrromethene 597

**Figure 4:**
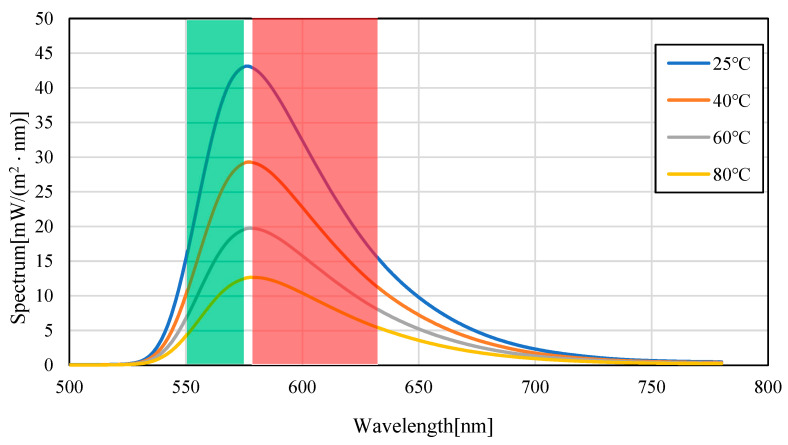
Fluorescence spectrum of Pyrromethene 597 in silicone oil. (Filled-in area: wavelength band of Red/Green images).

**Figure 5:**
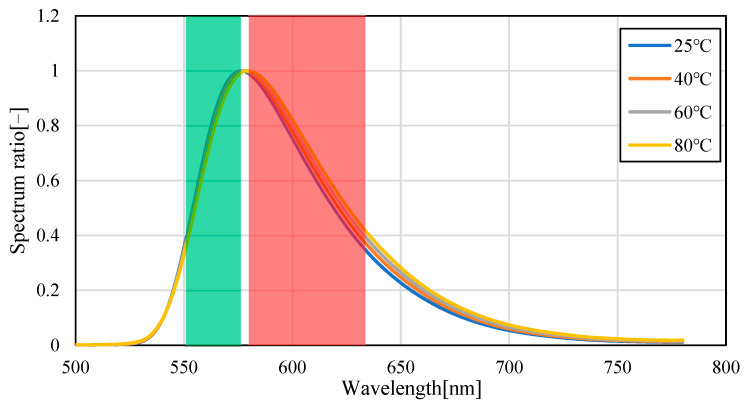
Fluorescence spectrum of Pyrromethene597 in silicone oil (normalized). (Filled-in area: wavelength band of Red/Green images).

**Figure 6:**
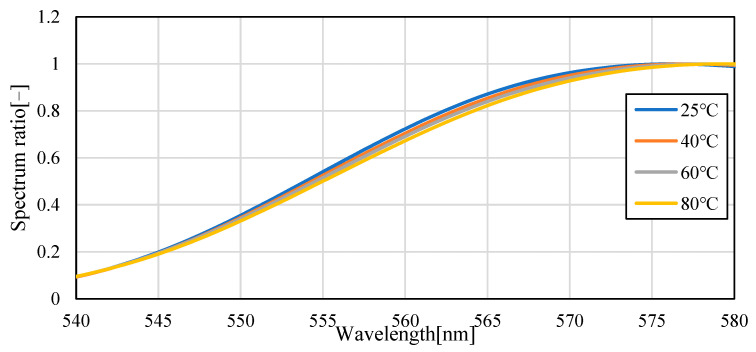
Fluorescence spectrum of Pyrromethene 597 in silicone oil (enlarged: 540–580 nm).

**Figure 7:**
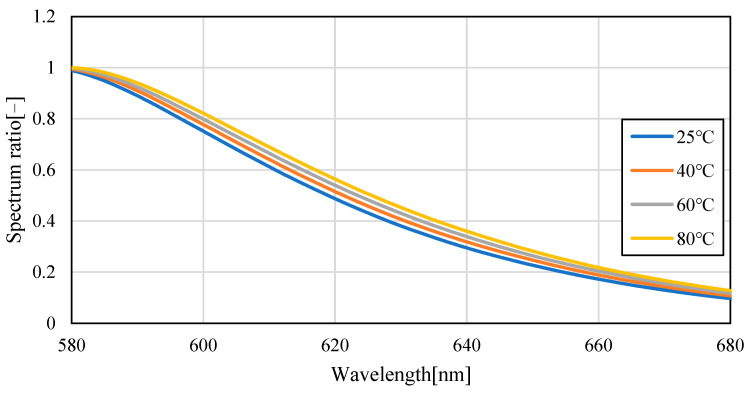
Fluorescence spectrum of Pyrromethene 597 in silicone oil (enlarged: 580−680 nm).

Figure 4 shows the emission spectra (smoothed by ±5 nm) of silicon oil mixed with Pyrromethene 597 at 25, 40, 60, and 80 °C. As shown in Figure 4, the absolute value of the spectrum decreased with increasing temperature. This is because of the decrease in the quantum yield of the phosphor with increasing temperature. A decrease of 70.6% was observed in fluorescence intensity when comparing the intensity of the maximum fluorescence wavelength from 25 °C to 80 °C, indicating that the camera conditions during image acquisition should be set to account for this decrease in brightness. Focusing on the change in the fluorescence wavelength, a graph normalized by the maximum spectral intensity is shown in Figure 5, which shows that the spectrum changes from 576 nm to 579 nm as the temperature increases. Comparing Figure 6, which focuses on the spectrum before the maximum fluorescence wavelength, and Figure 7, which focuses on the spectrum after the maximum fluorescence wavelength, there was a 4.36% decrease in the area ratio between 25 °C and 80 °C from 500 nm to 577 nm, and the area fraction at 80 °C increased by 12.84% from 578 nm to 700 nm. The large variation at wavelengths longer than the maximum fluorescence wavelength indicates that temperature variation occurs as a distribution in the fluorescence spectrum, rather than as a redshift of the fluorescence wavelength.

**Figure 8:**
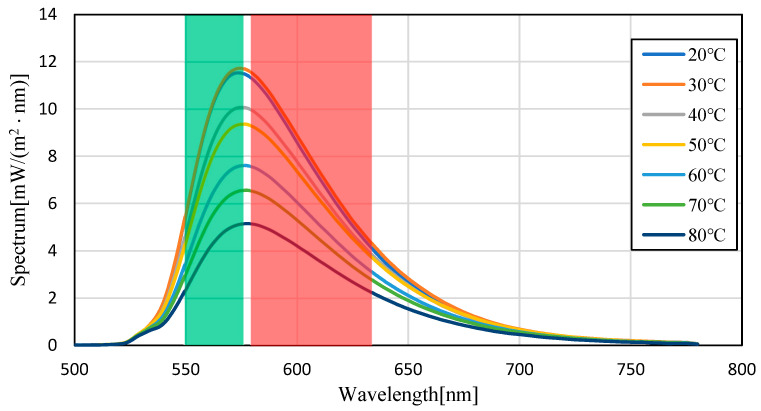
Fluorescence spectrum of Pyrromethene 597 in transmission oil. (Filled-in area: wavelength band of Red/Green images).

**Figure 9:**
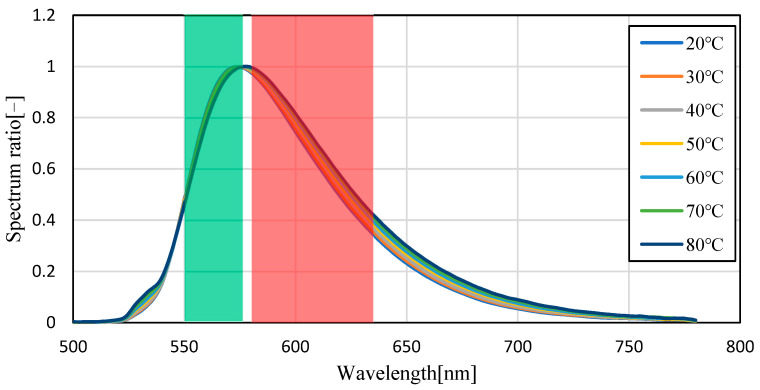
Fluorescence spectrum of Pyrromethene 597 in transmission oil (normalized). (Filled-in area: wavelength band of Red/Green images).

**Figure 10:**
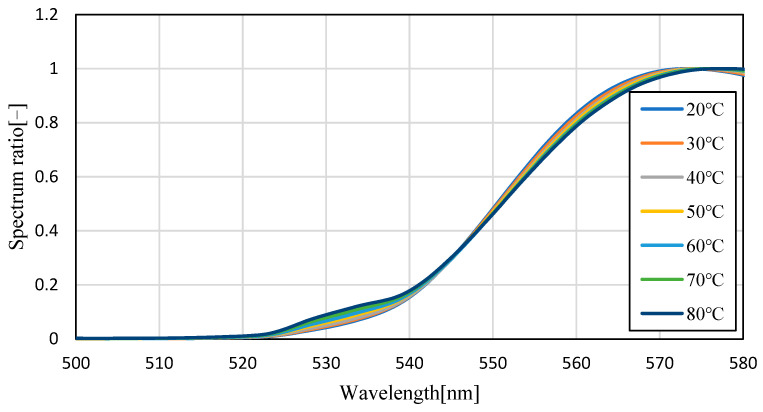
Fluorescence spectrum of Pyrromethene 597 in transmission oil (enlarged: 500–580 nm).

**Figure 11:**
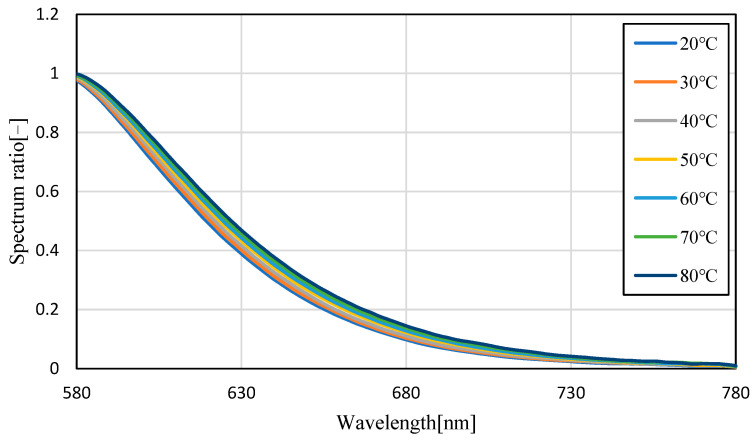
Fluorescence spectrum of Pyrromethene 597 in transmission oil (enlarged: 580–780 nm).

Figure 8 and Figure 9 show the fluorescence spectra at every 10 °C within the range of 20–80 °C in transmission oil mixed with Pyrromethene 597 at a concentration of 10.77 mg/L, and the graphs normalized by the maximum fluorescence wavelength, respectively. Figure 10 focuses on the spectrum before the maximum fluorescence wavelength. Figure 11 focuses on the spectrum after the maximum fluorescence wave-length. Figure 8 shows that a decrease in spectral intensity occurred owing to temperature quenching, similar to the results for silicon oil. Figure 9 shows that the maximum fluorescence wavelength varied from 573 nm to 578 nm, which is similar to the results obtained in the experiment using silicone oil as a solvent in the transmission oil. The graphs for before and after the maximum fluorescence wavelength were compared with those obtained for silicon oil. This was because of the excess excitation light that could not be absorbed by the phosphor and was reflected by thermal quenching, and the intensity at approximately 532 nm was increased. The percentage of the area after the maximum fluorescence wavelength increased by 15.45%, which was a larger change than that observed for silicon oil.

Several factors affect the fluorescence wavelength of a phosphor: the composition of the phosphor molecules and the properties of the solvent are assumed to have a large influence on the fluorescence wavelength. Among them, the change in the fluorescence wavelength of Pyrromethene 597 in the silicon and transmission oils used in this study is assumed to be because of the elongation of the Pyrromethene 597 molecule caused by the increase in the carbon content of the solvent oil, resulting in a red shift in the absorption and fluorescence wavelengths [19]. In addition, the viscosity of the solvent decreased with increasing temperature, which increased the rotational speed of the molecules, and the energy of the phosphor was released from the analysis area to outside the tank via heat [19]. In future studies, we plan to conduct a more detailed analysis using optical sensors [20] and attempt to optimize the slope value by obtaining better temperature characteristics.

**Figure 12:**
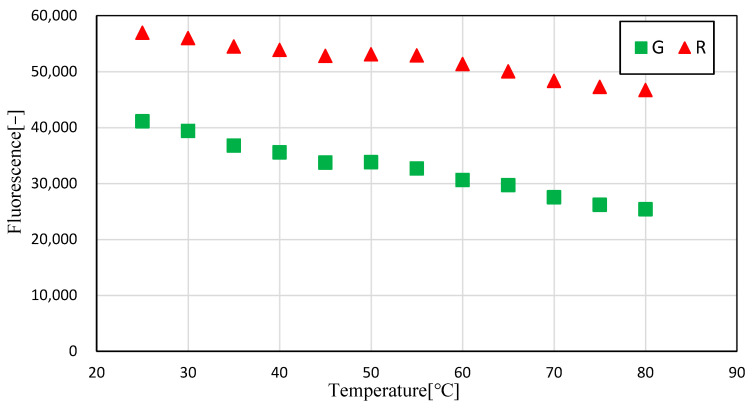
Relationship between temperature and intensity (Green/Red).

Figure 12 shows the results obtained from calculating the average luminance of the G and R images at each temperature. The results show that the luminance of both G and R decreased with increasing temperature, and that the rate of decrease of R was higher than that of G.

**Figure 13:**
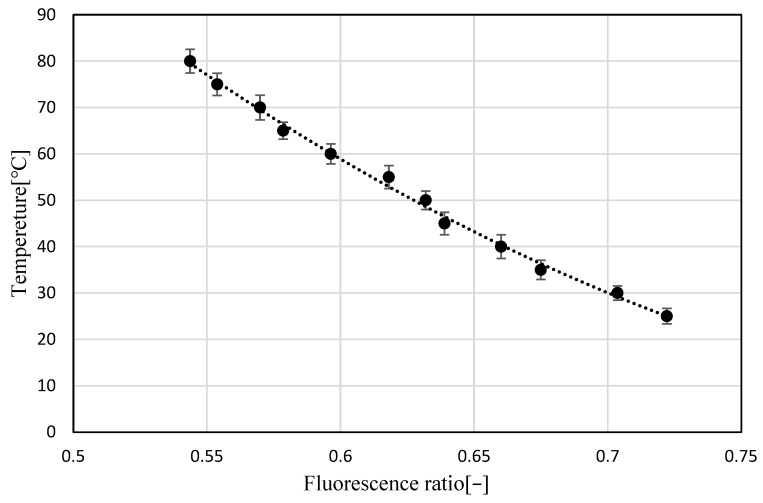
Relationship between temperature and Green/Red.

Figure 13 shows the relationship between the luminance ratio (G/R) and temperature using the calculated G and R luminance values, as shown in Figure 12. The results show that the luminance ratio increased with the temperature of the transmission oil containing Pyrromethene 597. These results show that the temperature dependence of the brightness ratio (fluorescence intensity ratio) in transmission oil with Pyrromethene 597 can be confirmed and that the LIF method can be used to measure the temperature.

### 3.2. Uncertainty Analysis

The total uncertainty in temperature is defined by the following equation:

(8) $$T_{c}^{2}\left(x\right)=\sum_{i=1}^{N}T_{}^{2}\left(x_{i}\right)$$

where *N* denotes the number of uncertainty factors. In other words, using multiplication, *T(x_i_)* is the uncertainty factor. The uncertainty originated from a thermocouple (±0.05 K bias error) and the non-uniform temperature field.

To evaluate the accuracy of temperature detection in the analysis area, the temperature distribution was measured every 5 K within the range of 25–80 °C, and the standard deviations were calculated for the temperature data at all coordinates (Table 1). The results indicate that the average accuracy was 2.19 K (precision error). The decrease in the measurement accuracy with increasing temperature can be attributed to factors such as temperature quenching. Using Equation (8), the composite uncertainty was evaluated as ±2.19 K.

## 4. Application to Flow Fields

**Figure 14:**
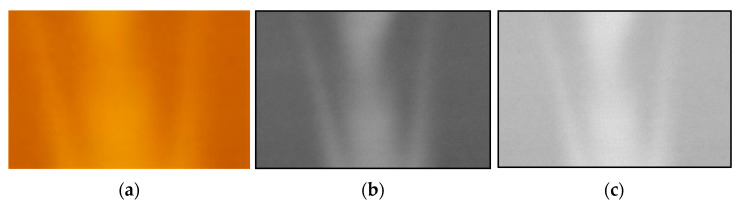
Visualized image of the vertical buoyant plume. (**a**) Color image; (**b**) Green channel; (**c**) Red channel.

The validity of the temperature distribution measurement method was evaluated by applying the two-color LIF method with Pyrromethene 597 to a buoyant jet flow. The transmission oil in the tank was maintained at a high temperature (80 °C) and a low temperature (25 °C), and was poured from the top of the tank. Consequently, a jet stream descending through the tank was generated. An image of the buoyant jet is shown in Figure 14a.

The process of temperature distribution measurement conducted in this study is described below.

**Figure 15:**
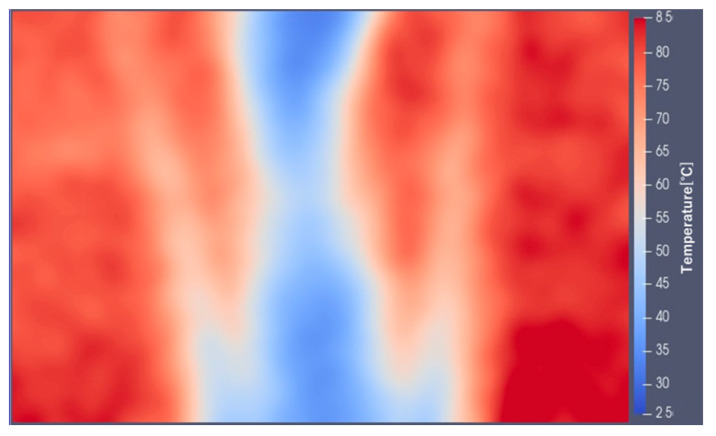
Temperature distribution of the vertical buoyant plume.

(1) The analysis area of the acquired R and G images was cropped and converted into a grayscale image.
(2) Noise was removed from the analyzed images.
(3) The luminance value of each pixel in the denoised image was expressed as a numerical data distribution:
(4) The distribution of luminance data for each of the R and G images was calculated (Figure 14b,c) using the numerical values of the corresponding coordinates to obtain G/R.
(5) The approximate curve obtained in Figure 13 was applied to the luminance ratio distribution to obtain the temperature distribution data.
(6) The temperature distribution data were converted into a two-dimensional color map (Figure 15).

Figure 14b,c are grayscale G and R images of the analysis area. The temperature distribution typical of plume phenomena generated by buoyant jets is also shown in Figure 15. The temperature distributions obtained from the measurements were found to be consistent with the results of research on plume phenomena [21]. It was confirmed that this measurement method is effective for measuring the temperature distribution of transmission oil. In addition, the use of Pyrromethene597 and the two-wavelength LIF method showed that the effect of laser light attenuation was not a problem. Therefore, there is a promising prospect that this measurement method can be maintained even for transmission oil flows with bubbles caused by cavitation phenomena. In addition, the temperature distribution of the transmission oil around the bubbles was measured with a high magnification microscope, and it was demonstrated that the oil around the bubbles could be clearly visualized and photographed.

## 5. Conclusions

A two-color LIF method using Pyrromethene 597 was developed, its temperature measurement accuracy was evaluated, and it was applied to buoyant jet phenomena. As a result, the following conclusions were obtained.

The maximum fluorescence wavelength of the transmitted oil mixed with Pyrrome-thene 597 varied with temperature. The emission ratio at longer wavelengths decreased compared to the emission ratio of the maximum fluorescence. This fluorescence characteristic was similar for transmission oil mixed with Pyrromethene 597, confirming the existence of a dependence between the fluorescence intensity ratio (G/R) and temperature. To validate the effectiveness of this measurement method, it was applied to buoyant jet flow phenomena, and the temperature distribution was visualized by the two-wavelength LIF method using the change in the fluorescence spectrum of one fluorophore. The temperature distribution was visualized every 10 °C within the range of 30 to 80 °C, and the mean standard deviation was 2.24 °C. The results of the visualization measurements demonstrate the validity of this measurement method and provide the prospect of verifying the actual temperature change in transmission oil due to cavitation with micrographs.

## Figures and Tables

**Table 1:** Temperature measurement accuracy at each measurement.

Temperature, °C	25	30	35	40	45	50	55	60	65	70	75	80
Standard deviation, K	1.68	1.52	2.07	2.56	2.41	1.98	2.47	2.16	1.82	2.66	2.37	2.57

## Data Availability

Not applicable.
